# From Biowaste to Lab-Bench: Low-Cost Magnetic Iron Oxide Nanoparticles for RNA Extraction and SARS-CoV-2 Diagnostics

**DOI:** 10.3390/bios13020196

**Published:** 2023-01-28

**Authors:** Le Yu, Penelope Adamson, Pei Lay Yap, Tran Tung, Shaheer Makar, Mark Turra, Geoff Higgins, Dusan Losic

**Affiliations:** 1 School of Chemical Engineering and Advanced Materials, University of Adelaide, Adelaide, SA 5005, Australia; 2 ARC Hub for Graphene Enabled Industry Transformation, The University of Adelaide, Adelaide, SA 5005, Australia; 3 SA Pathology, Adelaide, SA 5000, Australia; 4 Faculty of Pharmacy, Assiut University, Assiut 71526, Egypt

**Keywords:** SARS-CoV-2, real-time polymerase chain reaction (RT-PCR), RNA extraction, magnetic iron-oxide nanoparticles, biowaste, bacterial biofilm

## Abstract

The gold standard for diagnostics of SARS-CoV-2 (COVID-19) virus is based on real-time polymerase chain reaction (RT-PCR) using centralized PCR facilities and commercial viral RNA extraction kits. One of the key components of these kits are magnetic beads composed of silica coated magnetic iron oxide (Fe_2_O_3_ or Fe_3_O_4_) nanoparticles, needed for the selective extraction of RNA. At the beginning of the pandemic in 2019, due to a high demand across the world there were severe shortages of many reagents and consumables, including these magnetic beads required for testing for SARS-CoV-2. Laboratories needed to source these products elsewhere, preferably at a comparable or lower cost. Here, we describe the development of a simple, low-cost and scalable preparation of magnetic nanoparticles (MNPs) from biowaste and demonstrate their successful application in viral RNA extraction and the detection of COVID-19. These MNPs have a unique nanoplatelet shape with a high surface area, which are beneficial features, expected to provide improved RNA adsorption, better dispersion and processing ability compared with commercial spherical magnetic beads. Their performance in COVID-19 RNA extraction was evaluated in comparison with commercial magnetic beads and the results presented here showed comparable results for high throughput PCR analysis. The presented magnetic nanoplatelets generated from biomass waste are safe, low-cost, simple to produce in large scale and could provide a significantly reduced cost of nucleic acid extraction for SARS-CoV-2 and other DNA and RNA viruses.

## 1. Introduction

Since the emergence of the SARS-CoV-2 coronavirus in 2019, infection has spread around the globe to become a pandemic disease with more than 600 million people infected resulting in ca. 6.5 million deaths [1,2]. Many strategies applied in response to pandemic emergencies aim to provide reliable, low-cost and available tools for effective diagnostics [3]. To date, several low-cost diagnostic methods using enzyme-linked immunosorbent assay (ELISA), colorimetric oligonucleotides assays, electrochemical or optical biosensors using nucleic acid, antibody and aptamers, were explored [4,5]. However, only a few were commercialized and are currently used for fast and low-cost testing of COVID-19 infection [4,5,6]. While the use of these portable devices, such as rapid antigen testing, is useful for fast and initial screening, the real-time polymerase chain reaction (RT-PCR) method is still recommended as the gold standard methodology for in vitro diagnostics of SARS-CoV-2. The RT-PCR is a routine method that has been widely used in research and bio-diagnostics over the last 30 years to detect genetic information from biological samples, and this method has been quickly developed for use as the primary method for SARS-CoV-2 detection [6,7]. The PCR-based method has some disadvantages such as a lack of portability, the use of centralized and expensive lab facilities with high infrastructure and capital cost, experienced and trained personal needed to conduct testing, and high operational costs using expensive extraction kits and reagents that limit the delivery of cost-efficient diagnostics of SARS-CoV-2 [8,9,10]. 

More than 50 RT-PCR kits from different companies (such as, Thermo Fisher Scientific Inc., MA 02451 USA, Roche Molecular Systems Inc., Pleasanton, CA 94588, USA, Quest Diagnostics, Secaucus, NJ 07094 USA, Abbott Molecular Des Plaines, IL 60018, USA, Promega, Madison, WI, USA). A with approval by the World Health Organization, or by national health authorities, are currently commercially available for RNA extraction and SARS-CoV-2 detection [10,11,12,13]. These kits are used to extract and amplify regions of the virus’ genetic material, usually targeting the E and N genes of SARS-CoV-2. The viral RNA is reverse-transcribed to DNA, and then amplified using repeated temperature cycles in a PCR machine and fluorescent markers used to indicate the presence of the virus in the patient sample [14]. At various times throughout the pandemic, but particularly in the beginning, there was a supply shortage of many reagents and consumables required for SARS-CoV-2 testing due to their increased demand around the world. This was particularly critical in less developed countries with limited access to these reagents and PCR equipment. One of the key components from these kits are the magnetic beads composed of silica coated iron oxide (Fe_3_O_4_ magnetite). These magnetic beads were used in PCR analysis to selectively bind genetic materials (RNA or DNA), which are separated from the rest of biological sample by magnet and used for further processing (amplification and quantification) [10]. The market and supply of these kits and magnetic nanoparticles are dominated by USA biotech companies such as Thermo Fisher Sci. (MA 02451 USA USA), Luminex Co (USA) Spherotech Inc. (Libertyville, Illinois, USA) Bangs Laboratories (IN 46038, USA); Promega (Madison, WI, USA) [10,14,15]. The high price for silica coated MNPs, in the range of USD 150–200 for 5–10 mL of 100 mg/mL particle solution has considerable impact on the cost of qPCR testing of SARS-CoV-2 and other viral and bacterial infections. These magnetic nanoparticles are usually prepared by several synthetic methods, such as high-temperature thermal decomposition and/or reduction, coprecipitation, hydrothermal synthesis and sono-chemical synthesis [15,16]. One of the main problems with these production methods is their low throughput, lack of scalability and high manufacturing cost making these reagents expensive and with limited supply. Additionally, most of these methods can produce only spherical MNPs, which are only used for PCR, RNA and DNA extraction. Some of these preparation methods are proven to generate other shapes such as nanorods, nanowires and nanoflowers, which can be advantageous for these extractions but surprisingly not explored for these applications. Alternative methods, such as electrochemical synthesis and laser pyrolysis techniques, have been recently explored to improve the production capacity and lower costs that could potentially diversify the supply of MNPs and contribute to lower the operational cost of SARS-CoV-2 diagnosis [16,17,18].

**Figure 1:**
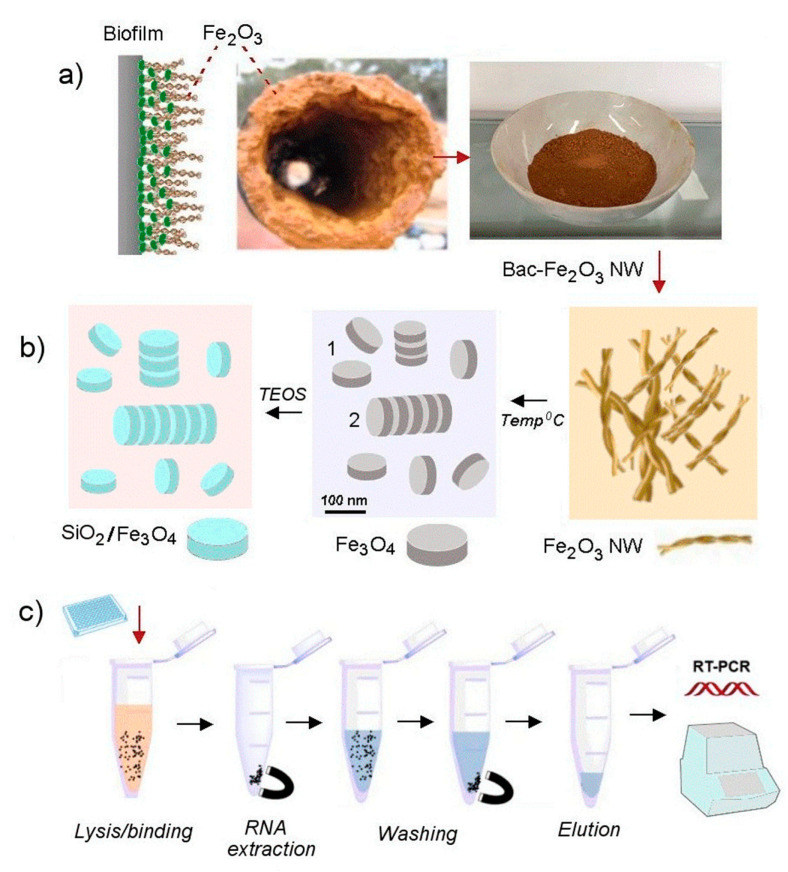
Schematic diagram of preparation of low-cost iron oxide magnetic nanoparticles (nanoplatelets) from bacterial biofilm (Bac-Fe_2_O_3_ nanowires) and their application for RNA extraction and SARS-CoV-2 RT-PCR: (**a**) the growth of biofilm in water pipes by iron oxidizing bacteria creating biowaste rich with bacterial iron oxide nanowires (Bac-Fe_2_O_3_ NW); (**b**) conversion of collected biowaste Bac-Fe_2_O_3_ NW into magnetic nanoparticles (Fe_3_O_4_) with nanoplate and nanorod shapes by thermal treatment following their coating with ultra-thin silica layer (SiO_2_-Fe_3_O_4_); and (**c**) the application of silica coated magnetic nanoplatelets for RNA extraction followed by SARS-CoV-2 RT-PCR.

This paper presents a simple and low-cost method to produce MNPs from biowaste, to address the cost and production scalability limitations of commercial MNPs needed for biomedical applications and demonstrate their successful use for RNA extraction and qPCR for SARS-CoV-2. The preparation method of these MNPs is based on the conversion of natural biowaste composed of biofilm generated in water pipelines by iron-oxide producing bacteria [19,20,21,22]. It is well-known that several iron oxidizing bacteria such as *Mariprofundus ferrooxydans* have the capability to produce iron-oxide biofilms from iron rich waters which usually occur in transporting water pipelines [23]. So far in our previous work, we have demonstrated how these biofilms containing iron-oxide nanowires, can be transformed into magnetic Fe_3_O_4_ nanoparticles with different shapes such as wires, rods and plates, and used for several applications such as adsorption of water contaminants, drug delivery and sensing [19,20,21,22,24,25]. In this paper, to demonstrate the application of these MNPs for RNA extraction and RT-PCR detection of SARS-CoV-2, we used Fe_3_O_4_ nanoparticles with nanoplate shapes. Figure 1 shows the preparation of these magnetic nanoplates from biowaste, followed by silica coating, nucleic acid extraction and SARS-CoV-2 PCR. It was proposed that these two-dimensional (2D) magnetic nanoplates would have an advantage over commercial spherical magnetic beads with 0-D structure due to their higher surface area for molecular assembly and better dispersion in water solutions. 

## 2. Materials and Methods

### 2.1. Materials and the Preparation of Magnetic Nanoplatelets (Fe_3_O_4_)

Tetraethyl orthosilicate (TEOS) and aqueous ammonia hydroxide (NH_4_OH, 28%) were purchased from Sigma-Aldrich (Sydney, Australia). Milli-Q water (18.2 MΩ.cm, Option-Q, Purelabs, Sydney, Australia) was used to prepare all the solutions used throughout this study. All chemicals are of analytical reagent grade and were used without further purification. 

Bacterial biofilm waste was provided by SA Water (South Australia). The waste material, after washing with water, was annealed at high temperature at 600–800 °C for 2 h. Resulting powders with MNPs in the form of nanoplates were cooled down and stored for further silica modification using a procedure adapted from Dayana et al. [26]. To make silica-coated MNPs, the prepared material was dispersed by sonication for 1 h in aqueous media. Typically, 1 g MNP powder was dispersed in 200 mL MilliQ-water, then 10 mL ammonia solution (28%) was added dropwise to the dispersion for 30 min under stirring in ambient conditions, followed by dropwise addition (ca 10 mL) of TEOS/ethanol solution (50%) to the mixture for 20 min to form a milky suspension. The product was then collected and purified by centrifugation (1976 RCF, 15 min) several times with water and ethanol, and dried in a vacuum oven for 12 h to produce silica-coated MNPs. Finally, MNP powder was dispersed in nuclease-free water at a concentration of 100 mg/mL and for DNA/RNA extraction.

### 2.2. Structural and Chemical Characterizations

High resolution scanning electron microscopy (HRSEM, FEI Quanta 450 FEG-SEM, USA) was used to study the morphology of the prepared materials. Transmission electron microscopy (TEM) characterization was performed using FEI Tecnai Bio Twin at 120kV by preparing samples on standard copper grids with a carbon support film. Obtained images were processed by Image J software and provided particles dimension were based on average numbers plus standard deviation made from at least 20 particles. All the prepared samples were scanned at 10 ° min^−1^ from 2θ = 5 to 80° using X-ray diffraction (XRD, Rigaku MiniFlex 600, Japan) at 40 kV. Fourier-transform infrared (FTIR) spectroscopy (Nicolet, 6700 Thermo Fisher, Australia) was adopted to detect the functional groups in the samples from 500 to 4000 cm^−1^ wavelength, while Raman spectroscopy (LabRAM HR Evolution, Horiba Jvon Yvon Technology, Japan) using 532 nm laser as the excitation source in the range of 0–2000 cm^−1^ was applied to distinguish the type of iron oxides. The thermal properties of the prepared samples were probed using TGA/DSC2 (STARe System, Mettler Toledo, Switzerland) under air atmosphere with the sample heated in an alumina crucible to 1000 °C at the heating rate of 10 °C min^−1^. Particle size distribution (PSD) was determined using water as the dispersant through dynamic light scattering (DLS) technique using Zetasizer Nano (Malvern Analytical Australia, Australia). The sample was dispersed in deionized water for PSD measurement. Colloidal stability of prepared dispersions was performed qualitatively by their visual observations in small vials over the time. If no segregation over 24 h is observed sample is regarded to have a good stability. The zeta potential of the sample was measured as a function of pH in triplicate using a Malvern Zetasizer (Nanoseries, Australia). The sample was first dispersed in Milli-Q water with the pH of the mixture adjusted in the range of 3–11 using HCl or NaOH solution in a clear disposable zeta cell and allowed to equilibrate for at least 120 s prior to zeta potential measurement. The concentration of the prepared sample was between 0.1–1 *w*/*v* % to achieve at least 20 kcps (kilocounts (of photons) per second) count rate with the dispersant (water) refractive index (RI) at 1.330. 

### 2.3. Nucleic Acid Extraction and RT-PCR Detection of SARS-CoV-2 

SARS-CoV-2 positive control (VIDRL, Victoria, Australia) was produced from a cell lysate, gamma irradiated and stored in Sigma Virocult (VTM; Medical Wire and Equipment, Corsham, England) at −80 °C. 2 µL of SARS-CoV-2 positive control stock was used to inoculate 2 mL VTM. The final concentration of the positive control was designed to achieve a 30 positive result (cycle threshold (Ct) 28–30) in an in-house COVID-19 RT-PCR assay. VTM without the addition of SARS-CoV-2 was used as a negative control.

Magnetic particles were resuspended in DEPC-treated (nuclease-free) water (Ambion, Thermo Fisher Scientific, Waltham, MA, USA) at 100 mg/mL and were stored at room temperature. Nucleic acids were extracted from the control using the Applied Biosystems by Thermo Fisher MagMAX™ Viral/Pathogen Kit (Thermo Fisher Scientific, Adelaide, Australia). Magnetic particles from the kit were used as the reference for extraction and were replaced with the experimental particles at the same concentration as the particles from the kit for magnetic particle assessment. Extractions were carried out as described in the manufacturer’s protocol. Briefly, 200 µL of the spiked VTM was added to a lysis plate well containing 530 µL binding solution, 2 mg magnetic particles and 22 µL Multi-IC/polyA (in-house internal control and carrier RNA). Extractions were performed on a KingFisher Flex (Thermo Fisher Scientific) using a script (MVP_Std) provided by Thermo Fisher Scientific. Magnetic particles were washed with 1 mL wash buffer, followed by 1 mL 80% ethanol (Chem-supply, SA, Australia) and then again with 500 µL 80% ethanol. Samples were eluted in 100 µL elution buffer.

2.5 µL of eluted RNA was assayed in an in-house RT-PCR protocol based on literature by Corman et al. [27]) which detects the E gene of SARS-CoV-2 (510 channel, and an extraction/PCR internal control (580 channel) in a reaction mix containing each set of primers and probes, 2X SuperScript^®^ III Platinum^®^ One-Step qRT-PCR System reaction mix with SuperScript^®^ III RT/Platinum^®^ Taq Mix (Invitrogen by Thermo Fisher Scientific) in a 12.5 µL reaction which was cycled at 1 cycle of 50 °C for 15 min, 1 cycle of 95 °C for 10 min and 45 cycles of 95 °C for 10 s, 55 °C for 15 s and 60 °C for 30 s on a LightCycler^®^ 480 (Roche, Indianapolis, IN, USA).

## 3. Results and Discussions

### 3.1. Characterizations of Prepared Magnetic Iron Oxide Nanoplatelets (MNPs) from Biowaste 

**Figure 2:**
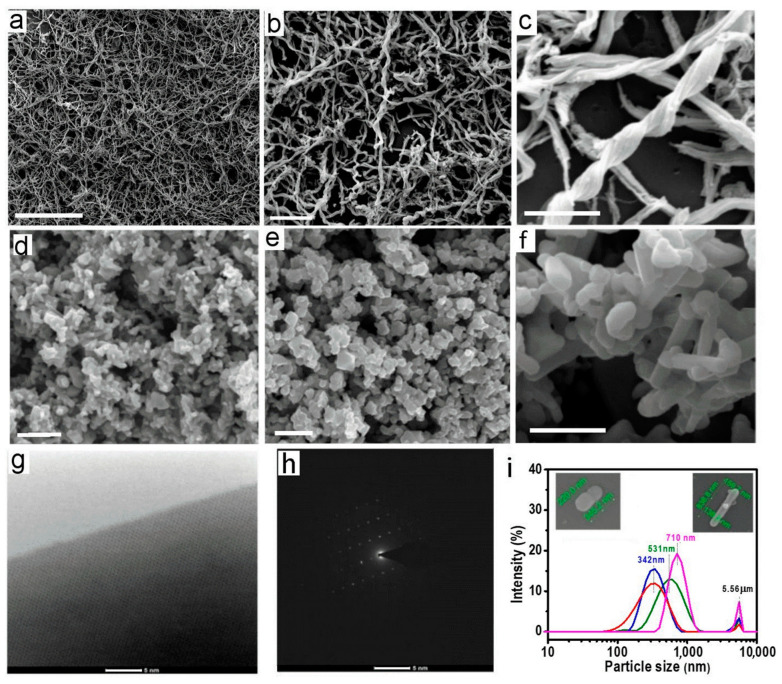
Scanning electron microscopy (SEM) images of (**a**–**c**) raw bacterial biofilm waste showing typical nanowire structures of iron oxide material (Bac-Fe_2_O_3_NWs) produced by bacteria which are converted by thermal treatment into; (**d**–**f**) magnetic Fe_3_O_4_ nanostructures with nanoplate (majority) and nanorod shapes; (**g**,**h**) High resolution TEM image and diffraction pattern image confirmed high crystalline structure of produced Fe_3_O_4_ particles; and (**i**) particle size distribution (PSD) from their dispersion show their size distribution in agreement with SEM results showing two typical shapes (bar scales for (**a**) 10 µm; (**b**) 5µm; (**c**) 500 nm; (**d**) 2 µm; (**e**) 1um and (**f**) 500 nm).

**Figure 3:**
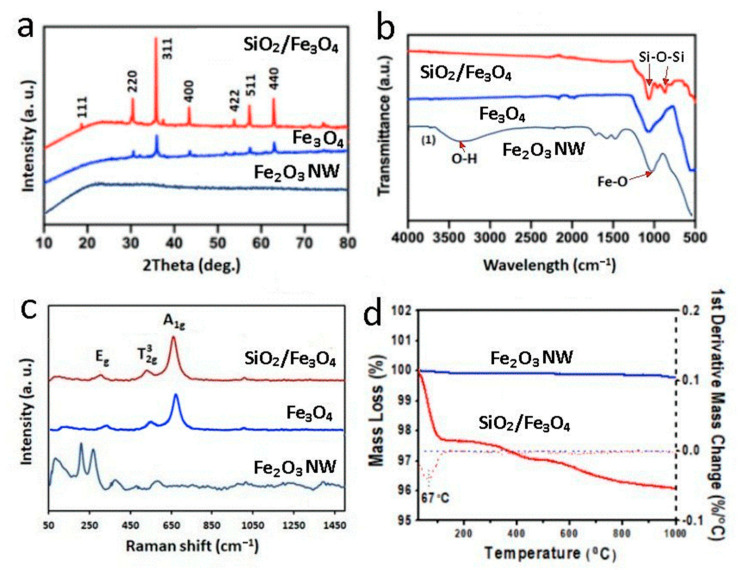
Comparative (**a**) XRD, (**b**) FTIR, (**c**) Raman and (**d**) TG-DTG of raw iron oxide nanowires (Bac-Fe_2_O_3_NWs) from bacterial film biowaste, followed by their thermal conversion into magnetic Fe_3_O_4_ nanoplatelets and further silica coating (SiO_2_/Fe_3_O_4_).

To confirm the structural, physical, chemical and interfacial properties of the biowaste material from biofilm in pipelines and prepared magnetic iron oxide nanoplates using this biowaste material, a series of characterizations were performed using a broad range of methods including TEM, EDAX, SEM, XRF, XRD, Raman, TGA, PSD and zeta potential (Figure 2 and Figure 3). The SEM images of raw biofilm material presented in Figure 2a–c revealed typical morphology of bacterial Fe_2_O_3_ structures composed of a dense network of coiled nanowires and their bundles. The typical length of these nanowires was 10–30 µm with a diameter of 100–130 nm with a helical and coiled morphology having at least 4–6 wires bundled together into a twisted structure. These structures are in concordance with structures observed in previous studies [19,20,21,22,26]. It is worth noting that the mechanism of how these iron oxide nanowires is created by iron oxidizing bacteria (*Mariprofundus ferrooxydans*) is still not fully understood. It is known that this process involves several types of bacteria contained within the biofilm and using the iron from water to produce energy through the oxidation of Fe^2+^ to Fe^3+^, which creates unique stalks composed of nanowires [23,28]. Chemical composition analysis by EDX and XRF of these nanowires confirmed that they are composed of iron oxide (Fe_2_O_3,_ hematite) with some impurities from other adsorbed materials such as Si, Mg, Ca, Na, K and C not removed by purification process [19,20,21]. Their XRD plots showed no peak, confirming the absence of a crystalline structure and the presence of a typical pattern of amorphous iron oxide (Fe_2_O_3_) [29]. 

HRSEM images of the created Fe_3_O_4_ structures after thermal treatment of the collected bacterial iron oxide nanowires from biowaste are presented in Figure 2d–f showing significant morphological changes. Two types of Fe_3_O_4_ nanostructures in two different shapes were observed. The first, nanoplatelets (nano discs), are dominant with 2-dimensional (2D) geometry, the second, nanorods, have 1D structure. The nanoplate structure had an average diameter of 300 ± 50 nm and dimensions for the nanorods were 140 ± 20 nm diameter and lengths ranging from 300 to 1400 nm. Both structures were observed to form aggregates as a result of drying during sample preparation for SEM imaging which is typical for magnetic nanoparticles. The prepared Fe_3_O_4_ nanostructures had very strong magnetic properties and high crystalline structure, which was confirmed by HRTEM and diffraction pattern image (Figure 2g,h). Particle size distribution (PSD) measurements from several different batches are presented in Figure 2i, confirming these dimensions for both forms. Both these shapes have higher specific surface area compared to the spherical structure of conventional magnetic beads used in the commercial viral nucleic acid extraction kits, which is advantageous as it provides more area for efficiently binding nucleic acids. For that reason, these structures were not separated as the mixture of 2D and 1D nanostructures is favorable for many applications compared with the spherical (0 D) shape of conventional magnetic beads.

More evidence confirming the successful transformation of raw bacterial iron-oxide (Fe_2_O_3_) nanowires from biowaste into crystalline magnetite Fe_3_O_4_ nanoplatelets showing their chemical and physical properties, is presented in Figure 3, with series of comparative XRD, FTIR, Raman and TG-DTG graphs. The XRD results in Figure 3a (Fe_2_O_3_ NW) show the typical signature of amorphous iron oxide for Bac-Fe_2_O_3_NWs after thermal annealing, converted to highly crystalline magnetic (Fe_3_O_4_), which confirmed successful conversion of bacterial iron-oxide nanowires. The XRD graphs with several major peaks at 2θ values of 30.2°, 35.6°, 38.1°, 43.2°, 53.5°, 57.2° and 62.8°, corresponding to the reflections of crystal planes (220), (311), (222), (400), (422), (511) and (440), respectively, which can be referenced to Fe_3_O_4_ (magnetite phase) [30]. These results can be explained by the reduction process of amorphous Fe2O3 into crystalline Fe_3_O_4_ structures which are reported in literature using both hydrothermal and thermal processes [31]. 

Meanwhile, the identity of magnetite was further supported by FTIR analysis (Figure 3b) with a strong transmission band at 570 cm^−1^ (marked by a red arrow), representing the Fe–O stretching mode of the tetrahedral site. A broad peak between 3000 cm^−1^ and 3500 cm^−1^ that corresponds to OH group confirmed the presence of iron oxyhydroxide type of Fe_2_O_3_. Raman spectroscopy has been recognized as a valuable tool to distinguish different phases of iron oxides [32,33]. The Raman spectrum of raw Bac-Fe_2_O_3_NWs sample, as depicted in Figure 3c, showed several characteristic bands of 219 cm^−1^, 283 cm^−1^, 398 cm^−1^ and 530.6 cm^−1^ of Fe_2_O_3_ (hematite). After annealing, the Raman spectrum showed several bands at 660.8 cm^−1^, 530.6 cm^−1^ and a weak band at 306.7 cm^−1^, which can be assigned to T_2g_ for Fe-O asymmetric stretching, A_1g_ for Fe-O symmetric stretching and E_g_ for Fe-O symmetric bending, respectively [33,34]. To evaluate the thermal stability and composition properties of the prepared magnetic nanoplatelets (Fe_3_O_4_) before and after silica coating, TGA analysis was carried out under air atmosphere from room temperature to 1000 °C (Figure 3d). From the TG-DTG plots, mass percentage of coated silica (SiO_2_) was calculated as about 1.75 mass percent. The flattened TGA curve of the uncoated Fe_3_O_4_ sample clearly indicated no obvious mass loss throughout the thermal treatment up to 1000 °C, suggesting it is thermally stable, without additional components, while the silica-coated sample showed significant mass loss with further details included in Section 3.2.

It Is important to state that the preparation of these magnetic Fe_3_O_4_ nanoplatelets is low-cost and based on a simple process using free biowaste, which is available in large quantities (tons). Using the simple lab batch process presented in this work, it is demonstrated that it is possible to make 1 kg of magnetic Fe_3_O_4_ within a few hours, without using toxic chemicals, which is the case for the conventional preparation of commercial magnetic nanoparticles, which use very long, expensive and laborious processes. 

### 3.2. Characterization of Prepared Silica Coated Magnetic Nanoplatelets 

**Figure 4:**
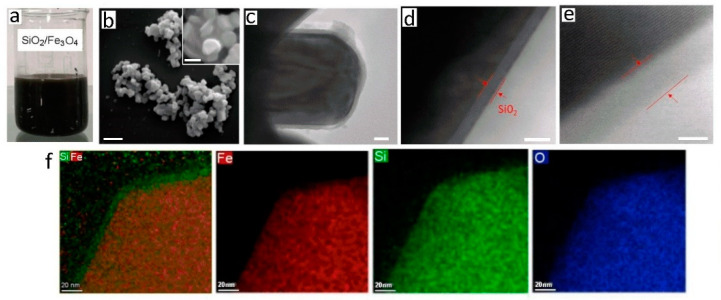
(**a**) Digital photograph showing dispersion of prepared silica coated magnetic nanoparticles (SiO_2_/Fe_3_O_4_); (**b**) SEM images after silica coating with inset showing their plate morphology in more details; (**c**–**e**) High resolution TEM images of single SiO_2_/Fe_3_O_4_ particles confirming the thickness of coated silica layer; and (**f**) series of TEM images of mapping of chemical composition confirming their elemental composition (Fe, O, Si). (Bar scale (**b**) 200 nm, inset 2 nm, (**c**–**e**) 2 nm).

To use prepared magnetic nanoplatelets for DNA/RNA extraction, silica coating of the particles was required. Silica coating is conventionally used for commercial magnetic nanobeads, providing desirable chemistry for binding nucleic acids. In this study, among several potential coating methods, TEOS was selected since it is known to produce a robust, thin, and repeatable coating. To demonstrate that silica coating on the magnetic nanoplatelets (Fe_3_O_4_) had occurred, comprehensive characterizations using XRD, FTIR, Raman, TGA, SEM, HRTEM and chemical mapping characterizations were performed before and after coating as summarized in Figure 3 and Figure 4. Comparative XRD analysis (Figure 3a) of uncoated and silica-coated Fe_3_O_4_ nanoplatelets showed only a negligible peak shift from 2θ = 35.9° (uncoated sample) to 35.7° (coated sample) on their respective reflection plane (311), which clearly augmented the crystalline structure of magnetite even after coating with silica [29]. FTIR results (Figure 3b) on the silica-coated samples showed two strong transmission bands: first at 868 cm^−1^ that can be attributed to the symmetric linear vibration of Si-O [5], and the second band at 1060 cm^−1^ represented the stretching vibration of Si-O-Si, indicating the successful silica coating on magnetite particles [4]. Meanwhile, all the Raman peaks found in the uncoated sample were also detected on the Raman plot of the coated sample, but with an additional band occurred at high frequency (1079.1 cm^−1^ (black arrow)) as shown in Figure 3c. This was as a result of the symmetrical stretching of silicon and oxygen in the silicate tetrahedral with non-bridging oxygen atoms. The typical Raman peaks between 400 cm^−1^ and 700 cm^−1^, representing the Si-O bonding and bending, were difficult to distinguish at the low frequency region of the Raman spectrum, since they can be easily overlapped by the strong peaks due to Fe-O bonding [33]. The TGA results (Figure 3d) reveal that silica coated Fe_3_O_4_ particles are relatively thermal-unstable compared to uncoated particles. A total mass loss of about 4.0% was observed on the coated sample with a significant mass loss below 100 °C which could be linked to the loss of water resulting from the silica coating process. 

Figure 4a show a good dispersion stability of 500 mL of a preparation of highly concentrated (200 mg/mL) SiO_2_/Fe_3_O_4_ dispersion that did not aggregate, which is common for magnetic nanoparticles. Zeta potential measurements (pH 3–11) did not show significant differences between uncoated and silica coated SiO_2_/Fe_3_O_4_ showing isoelectric point (IEP) values of 3,62 mV for coated and 4.13 mV for uncoated sample. SEM characterization (Figure 4b) showed no morphological differences between uncoated and silica coated magnetic nanoparticles, confirming very thin silica coating with TEOS with plate-like particles and a smooth surface that stacked up on one another. This observation was further verified by the PSD analysis determined by dynamic light scattering technique, showing a slight shift in particle size from before coating (Figure 2i), which is resulted from the additional silica layer on the surface of the Fe_3_O_4_ particles. To determine the thickness of this silica layer, HR-TEM with elemental mapping was applied (Figure 4c–f). The TEM images in Figure 4c–e reveal that a very thin and uniform silica layer of approximately 5 nm was created on the surface of the magnetic nanoplatelets. Chemical mapping images (Figure 4f) determined that this coating has Si, Fe and O chemical composition as proposed, which is satisfactory to provide the required silica binding chemistry.

**Figure 5:**
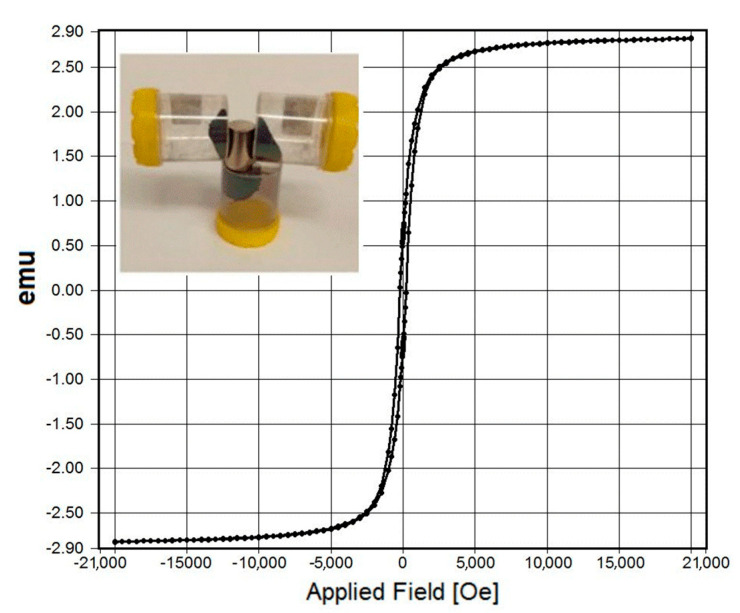
Typical magnetization curves of SiO_2_/Fe_3_O_4_ at room temperature with inset showing photo of strong attracting of SiO_2_/Fe_3_O_4_ powders by permanent magnet (neodymium).

Finally, the magnetic measurement of SPIO for silica modified Fe_3_O_4_ nanoplatelets (Figure 5) indicates superparamagnetic behavior at room temperature with no hysteresis and perfect Langevin behavior [27]. It is worth noting that no significant difference in the magnetic properties of the prepared Fe_3_O_4_ particles was seen after coating with silica showing overlapped magnetization curves. The excellent magnetic properties of prepared SiO_2_/Fe_3_O_4_ powders and dispersion were demonstrated using a permanent magnet (neodymium) and showed very high attraction confirming their practical applications for use in nucleic acid extraction.

### 3.3. Comparative Performance of Silica Coated Magnetic Nanoplatelets for RNA Extraction and SARS-CoV-2 RT-PCR

**Figure 6:**
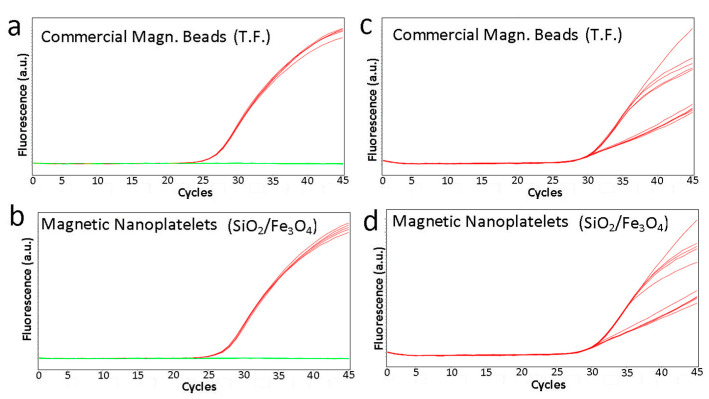
Silica coated magnetic nanoparticles perform comparatively to reference commercial magnetic particles for viral nucleic acid extraction. Comparative amplification curves of (**a**,**c**) reference commercial magnetic beads and (**b**,**d**) silica coated magnetic nanoparticles (SiO_2_/Fe_3_O_4_) for viral nucleic acid extraction as seen by E gene amplification (**a**,**b**) and internal control (**c**,**d**) in an in-house RT-PCR. Red curves indicate amplification of the E gene or the internal control target. Green lines (negative control VTM) indicate no amplification. The subset of flatter internal control curves corresponds to the SARS-CoV-2 positive samples and demonstrates competitive inhibition of the internal control PCR by the E gene amplification.

The substitution of the commercial preparation of magnetic particles from the Applied Biosystems by Thermo Fisher MagMAX^TM^ Viral/Pathogen Kit with the silica coated magnetic nanoplatelets demonstrated no difference in SARS-CoV-2 extraction efficiency from positive control extraction material (Figure 6a–d). Grouping of the five replicates for each magnetic particle type shows a slight shift to the left for the reference curves indicating slightly superior performance of the reference magnetic particles; however, the difference in Ct value is less than 0.5 of a cycle between the reference magnetic beads and the magnetic nanoplatelets (Table 1). 

## 4. Conclusions

In summary, this work presents the application of low-cost Fe_3_O_4_ magnetic nanoplate particles generated from bacterial biofilm waste for RNA extraction and RT-PCR. of SARS-CoV-2. These magnetic nanoparticles converted from bacterial iron-oxide nanowires (Fe_2_O_3_) showed a characteristic nanoplate shape which is proposed to be beneficial compared with the commercial spherical magnetic Fe_3_O_4_ particles produced by other methods and used for DNA/RNA extraction. The prepared magnetic Fe_3_O_4_ nanoplates were successfully coated with a thin silica layer which is required for RNA binding for their selective extraction. The SARS-CoV-2 RT-PCR results using the SiO_2_/Fe_3_O_4_ particles described here showed similar extraction capability to the commercially prepared magnetic beads in terms of sensitivity of virus detection, dispersion stability and workability. This work demonstrates that these magnetic SiO_2_/Fe_3_O_4_ particles obtained from biofilm with unique 2D shapes are a suitable substitute for commercial magnetic beads reagents. The production cost of these magnetic nanoparticles is very low, with a production capacity of 100 kg per batch scale, making them a suitable alternative to address the shortage of commercial magnetic beads material for DNA/RNA extraction kits needed for PCR diagnostics of SARS-CoV-2, or future emerging viruses and pathogens. 

## Figures and Tables

**Table 1:** Comparative results of silica coated magnetic nanoparticles (SiO_2_/Fe_3_O_4_) perform and the reference commercial magnetic beads for extraction of SARS-CoV-2 RNA from positive controls. Crossing point (Ct) values are an average of five replicates. T-test statistics was performed (Paired Two Sample for Means) was used to analyze obtained data to determine means, variance, correlation, P (T < = t) (one and two tail) and T critical (one and two tail).

Type of Magnetic Beads	SARS-CoV-2 Samples with E Gene Primers	SARS-CoV-2 Samples with IC Primers	Negative Samples with E Gene Primers	Negative Samples with IC Primers
**Ref. Magn. Beads**	26.75	27.79	0	30.27
	26.75	27.98	0	30.82
	26.75	27.85	0	30.71
	26.71	27.93	0	30.5
	26.84	28.03	0	30.3
**Average**	**26.752**	**27.916**		**30.52**
**Std Dev**	**0.053**	**0.096**		**0.243**
**Our SiO_2_/Fe_3_O_4_**	27.3	28.23	0	30.72
	27.36	28.09	0	30.65
	27.23	28.01	0	30.55
	27.04	28.12	0	30.62
	27.05	28.07	0	30.05
**Average**	**27.196**	**28.104**		**30.518**
**Std Dev**	**0.145**	**0.081**		**0.268**

## Data Availability

Not applicable.
